# Supplementary material for: Exercise Improves Myocardial Deformation But Not Cardiac Structure in Preterm-Born Adults: A Randomized Clinical Trial
Source: JACC Adv. 2025 Apr 28;4(5):101721. doi: 10.1016/j.jacadv.2025.101721 (PMC12063111; doi:10.1016/j.jacadv.2025.101721)
Supplement: Supplementary data [file mmc1.pdf]

Exercise Improves Myocardial Deformation but not Cardiac Structure in Preterm-born Adults: A Randomized Clinical Trial

Supplemental Tables

[1]Winok Lapidaire, PhD*; [1,2]Afifah Mohamed, DPhil*; [1,3]Wilby Williamson, DPhil; [1,4]Odaro J. Huckstep, DPhil; [1,5]Maryam Alsharqi, DPhil; [1,6]Cheryl M.J. Tan, DPhil; [7]Samuel Burden, PhD; [1,8]Cameron Dockerill, PhD; [1]William Woodward, BSc; [1]Annabelle McCourt, MSc; [1,9]Holger Burchert, DPhil; [1]Yvonne Kenworthy, BSc; [1]Luca Biasiolli, DPhil; [10]Helen Dawes, PhD; [11]Charlie Foster, PhD; [1]Paul Leeson, MB PhD; [12]Adam J. Lewandowski, DPhil**

**Joint first authors*

[1]Division of Cardiovascular Medicine, Radcliffe Department of Medicine, University of Oxford, Oxford, UK; [2]Faculty of Health Sciences, The National University of Malaysia, Kuala Lumpur, Malaysia; [3]School of Medicine, Trinity College Dublin, Dublin, Ireland; [4]Department of Biology, United States Air Force Academy, CO, USA; [5]Institute for Medical Engineering and Science, Massachusetts Institute of Technology, Cambridge, USA; [6]Ludwig Institute for Cancer Research, Nuffield Department of Medicine, University of Oxford, Oxford, UK; [7] Department of Women and Children's Health, King's College London, London, UK; [8]School of Biomedical Engineering and Imaging Sciences, King's College London, London, UK; [9]Department of Sport, Exercise and Health, University of Basel, Basel, Switzerland; [10]NIHR Exeter BRC, University of Exeter, Exeter, UK; [11]Centre for Exercise, Nutrition & Health Sciences, School of Policy Studies, University of Bristol, Bristol, UK; [12]Nuffield Department of Population Health, University of Oxford, Oxford, UK.

****Correspondence to:** Adam J. Lewandowski, BSc(Hons) MSt DPhil FESC
Nuffield Department of Population Health, Big Data Institute, Old Road Campus, University of Oxford, Oxford, UK OX3 7LF. Email: adam.lewandowski@ndph.ox.ac.uk

Supplemental Table 1: Baseline characteristics for participants allocated to the exercise intervention versus control groups

	**Exercise Intervention n=47**	**Control n=53**
Age, years	28.7 (4.1)	28.2 (4.5)
Male, n (%)	25 (53)	25 (47)
Employed, n (%)	44 (98)	49 (96)
University Degree, n (%)	35 (78)	43 (84)
Family history of cardiovascular disease, n (%)	21 (45)	19 (36)
BMI, kg/m^2	24.8 (3.4)	24.9 (3.5)
Smoker, n (%)	0 (0)	5 (10)
Units of alcohol per week	5.9 (5.5)	5.0 (7.5)
Awake ambulatory blood pressure, mmHg		
Systolic	129.5 (10.8)	129.0 (8.8)
Diastolic	77.4 (6.6)	77.9 (7.4)
Oxygen uptake peak, mL/kg/min	33.9 (7.2)	34.7 (7.2)
Cholesterol-HDL ratio	3.4 (1.0)	3.4 (1.2)
Glucose, mmol/L	4.8 (0.4)	4.9 (0.4)
HOMA insulin resistance	1.0 (0.5)	0.9 (0.4)

Values presented as mean (standard deviation) unless stated otherwise. BMI stands for body mass index; HDL, high density lipoprotein; HOMA, Homeostatic Model Assessment for Insulin Resistance.

Supplementary Table 2: Exercise intervention effect on cardiac magnetic resonance parameters

	Parameter	Exercise Intervention	Control	Adjusted mean difference (95% CI)	p-value
**Left Ventricle**					
Baseline	Ejection fraction, %	64.86 (5.41)	64.28 (6.26)	NA	NA
16 weeks		65.85 (6.17)	64.06 (5.61)	1.47 (0.13,2.82)	0.032
Baseline	Mass index, g/m^2	58.27 (8.68)	59.63 (9.3)	NA	NA
16 weeks		57.89 (9.03)	59.19 (9.48)	-0.22 (-1.51,1.07)	0.739
Baseline	End-diastolic volume index, mL/m^2	73.18 (8.9)	77.01 (14.19)	NA	NA
16 weeks		74.32 (8.82)	76.39 (12.93)	1.15 (-0.69,2.99)	0.217
Baseline	Stroke volume index, mL/m^2	47.4 (6.43)	49.36 (9.07)	NA	NA
16 weeks		48.96 (7.44)	48.71 (7.81)	1.8 (0.04,3.56)	0.045
Baseline	Length, mm	96.84 (8.25)	96.41 (9.26)	NA	NA
16 weeks		98.82 (7.69)	96.84 (9.29)	1.62 (0.82,2.41)	0
Baseline	Global Longitudinal Strain, %	-18.02 (2.58)	-18.69 (2.83)	NA	NA
16 weeks		-19.51 (2.43)	-19.16 (2.64)	-0.59 (-1.19,0)	0.05
Baseline	Basal Circumferential Strain, %	-17.84 (2.17)	-18.33 (2.19)	NA	NA
16 weeks		-19.01 (1.89)	-18.49 (2.21)	-0.79 (-1.29,-0.29)	0.002
Baseline	Mid Circumferential Strain, %	-18.5 (2.06)	-19.17 (2.16)	NA	NA
16 weeks		-19.69 (1.92)	-19.18 (2.11)	-0.82 (-1.45,-0.2)	0.01
Baseline	Apical Circumferential Strain, %	-20.74 (3.45)	-21.16 (3.35)	NA	NA
16 weeks		-21.55 (2.68)	-21.16 (2.86)	-0.68 (-1.36,-0.01)	0.047
**Right Ventricle**					
Baseline	Ejection fraction, %	59.07 (6.86)	58.17 (7.01)	NA	NA
16 weeks		61.58 (6.74)	59.18 (6.38)	1.91 (0.13,3.69)	0.035
Baseline	Mass index, g/m^2	19.06 (2.32)	18.47 (2.69)	NA	NA
16 weeks		18.77 (2.36)	18.74 (2.75)	-0.49 (-1.03,0.05)	0.076
Baseline	End-diastolic volume index, mL/m^2	79.26 (12.59)	81.69 (15.61)	NA	NA
16 weeks		82.08 (12.23)	80.79 (14.26)	3.39 (0.52,6.25)	0.021
Baseline	Stroke volume index, mL/m^2	46.56 (8.62)	47.52 (10.38)	NA	NA
16 weeks		50.51 (9.73)	47.6 (9.12)	3.8 (1.53,6.06)	0.001
Baseline	Global Longitudinal Strain, %	-20.93 (4.48)	-21.09 (4.08)	NA	NA
16 weeks		-22.33 (3.32)	-21.52 (3.85)	-1.12 (-1.88,-0.37)	0.004
**Aorta**					
Baseline	Ascending aorta distensibility, 10^{-3} mmHg^{-1}	6.35 (2.03)	6.57 (2.11)	NA	NA
16 weeks		6.56 (2.06)	6.55 (2.04)	0.12 (-0.51,0.76)	0.699
Baseline	Proximal descending aorta distensibility, 10^{-3} mmHg^{-1}	5.7 (1.68)	5.74 (1.5)	NA	NA
16 weeks		5.81 (1.52)	5.47 (1.28)	0.37 (-0.11,0.85)	0.128

Values presented as mean (SD) unless stated otherwise. P<0.05 are considered statistically significant.

Supplementary Table 3: Estimated bias by bootstrapping per analysis for term-born adults

Parameter	Original Estimate	Bootstrap Mean	Bias	95% BCa CI
**Structural cardiac parameters**				
LV mass index, g/m^2	-1.06	-1.04	0.02	(-3.02, 0.41)
LV end diastolic volume index, mL/m^2	1.88	1.81	-0.06	(-0.41, 4.13)
LV length, mm	2.09	2.07	-0.02	(0.98, 3.15)
LV mass/enddiastolic volume, g/mL	-3.43	-3.34	0.09	(-7.29, -1.34)
RV mass index, g/m^2	-0.14	-0.13	0.00	(-0.78, 0.44)
RV end diastolic volume index, mL/m^2	4.42	4.41	-0.01	(1.1, 7.95)
**Functional cardiac parameters**				
LV ejection fraction, %	1.87	1.87	0.00	(0.12, 3.67)
LV stroke volume index, mL/m^2	2.84	2.81	-0.03	(0.46, 5.23)
RV ejection fraction, %	2.86	2.81	-0.05	(0.6, 5.37)
RV stroke volume index, mL/m^2	5.53	5.51	-0.02	(2.83, 8.43)
**Myocardial deformation parameters**				
LV global longitudinal strain, %	-0.21	-0.32	-0.11	(-1.1, 0.92)
LV basal circumferential strain, %	-0.37	-0.39	-0.02	(-1.01, 0.22)
LV middle circumferential strain, %	-0.30	-0.30	0.00	(-1.22, 0.57)
LV apical circumferential strain,%	-0.46	-0.46	0.00	(-1.39, 0.48)
RV global longitudinal strain, %	-0.51	-0.54	-0.02	(-1.51, 0.54)

BCa CI stands for bootstrap bias-corrected and accelerated confidence interval.

Supplementary Table 4: Estimated bias by bootstrapping per analysis for preterm-born adults

Parameter	Original Estimate	Bootstrap Mean	Bias	95% BCa CI
**Structural cardiac parameters**				
LV mass index, g/m^2	0.78	0.74	-0.04	(-1.2, 2.98)
LV end diastolic volume index, mL/m^2	0.00	-0.03	-0.03	(-3.08, 2.32)
LV length, mm	0.91	0.88	-0.02	(-0.29, 1.73)
LV mass/enddiastolic volume, g/mL	1.56	1.57	<0.01	(-1.65, 4.75)
RV mass index, g/m^2	-0.87	-0.88	-0.01	(-1.75, 0.08)
RV end diastolic volume index, mL/m^2	1.60	1.59	-0.01	(-3.56, 6.75)
**Functional cardiac parameters**				
LV ejection fraction, %	0.78	0.79	0.01	(-1.06, 3.19)
LV stroke volume index, mL/m^2	0.22	0.19	-0.03	(-2.2, 2.51)
RV ejection fraction, %	0.88	0.88	-0.01	(-1.86, 3.15)
RV stroke volume index, mL/m^2	1.45	1.42	-0.03	(-2.43, 5.19)
**Myocardial deformation parameters**				
LV global longitudinal strain, %	-1.18	-1.21	-0.02	(-1.84, -0.29)
LV basal circumferential strain, %	-1.33	-1.34	-0.01	(-2.11, -0.53)
LV middle circumferential strain, %	-1.54	-1.55	<0.01	(-2.44, -0.67)
LV apical circumferential strain,%	-1.04	-1.05	-0.01	(-2.14, -0.08)
RV global longitudinal strain, %	-1.99	-2.02	-0.03	(-3.05, -0.97)

BCa CI stands for bootstrap bias-corrected and accelerated confidence interval.
